# Evaluation and management of adult idiopathic intracranial hypertension

**DOI:** 10.1136/practneurol-2018-002009

**Published:** 2018-08-28

**Authors:** Susan P Mollan, Catherine Hornby, James Mitchell, Alexandra J Sinclair

**Affiliations:** 1 Metabolic Neurology, Institute of Metabolism and Systems Research, University of Birmingham, Birmingham, UK; 2 Birmingham Neuro-Ophthalmology, Queen Elizabeth Hospital, Birmingham, UK; 3 Centre for Endocrinology, Diabetes and Metabolism, Birmingham Health Partners, Birmingham, UK; 4 Department of Neurology, University Hospitals Birmingham, Queen Elizabeth Hospital, Birmingham, UK

**Keywords:** idiopathic intracranial hypertension, headache, benign intracran hyp, neuroophthalmology, papilloedema

## Abstract

This paper summarises the first consensus guidelines for idiopathic intracranial hypertension as an infographic. Following a systematic literature review, a multidisciplinary specialist interest group met and established questions relating to population, interventions, controls and outcomes (PICO). A survey was sent to doctors who manage idiopathic intracranial hypertension (IIH) regularly. Statements were reviewed by national professional bodies, specifically the Association of British Neurologists, British Association for the Study of Headache, the Society of British Neurological Surgeons and the Royal College of Ophthalmologists and by international experts. Key areas are represented based on the guideline, namely: (1) investigation of papilloedema and diagnosis of IIH; (2) management strategies; and (3) investigation and management of acute exacerbation of headache in established IIH. We present an infographic as an aide-mémoire of the first consensus guidelines for IIH.

IIH is commonly associated with obesity, younger age and females.^1 2^ Patients present acutely to many different specialities and often have multiple acute visits through the course of their disease. The investigation and management of IIH is complex involving many specialities.^3^ This infographic summarises three key pathways based on the recommendations of a multidisciplinary, patient-involving and multiprofessional specialist interest group on the investigation and management of IIH.^4^

The basis of the specialist interest group included representation from neurology, neurosurgery, neuroradiology, ophthalmology, nursing, primary care doctors and patient representatives. Questions on PICO were defined and through a large Delphi group exercise; expertise was captured from a wide-reaching group of clinicians, thus reflecting practice from across the UK and internationally. The statements were then critically reviewed by key opinion leaders and by Association of British Neurologists, British Association for the Study of Headache, the Society of British Neurological Surgeons and the Royal College of Ophthalmologists. This is the first consensus guidance for optimal management of IIH.^4^

Identification of papilloedema can be challenging, and clinicians should be aware of the differential diagnosis of pseudopapilloedema (figure 1). Once papilloedema is confirmed, it requires urgent investigations, including lumbar puncture, where the patient experience could be greatly improved.^5^ Symptoms of IIH are not pathognomonic, and hence it is essential to apply the diagnostic criteria, including excluding secondary causes, for a definite diagnosis.^4^ The lumbar puncture opening pressure was one key area of debate. Within the wider Delphi group, it was clear that there is a ‘grey zone’ of lumbar puncture opening pressures between 25 cm cerebrospinal fluid (cmCSF) and 30 cmCSF, as to what each expert considered to be pathological, and this is reflected within the infographic thermometer for lumbar puncture opening pressure (figure 1).

**Figure 1:**
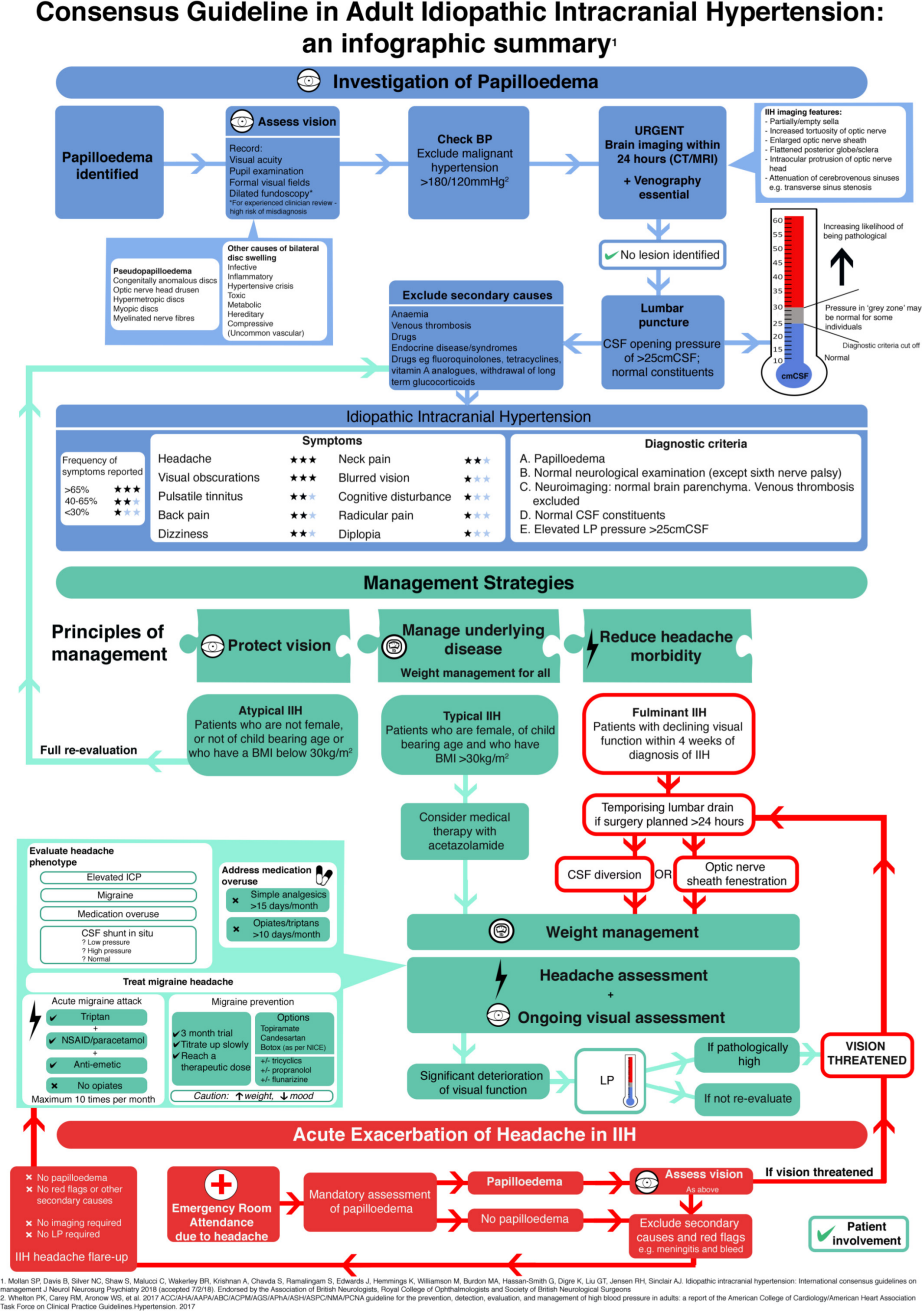
Consensus Guideline in Adult Idiopathic Intracranial Hypertension: an infographic summary.

Principles of management need to address both the rapidity of the disease that may lead to visual loss in some and require surgical intervention and the morbidity of the headache that can develop in the majority, which substantially affects the quality of life.^6^ Weight loss is currently the only established disease-modifying therapy^7^ and is notoriously difficult to achieve and maintain.

Evaluation of the headache phenotype is essential to target treatment and to help identify medication-overuse headache. Where there are features of migraine, topiramate may be the first line in treatment, and recent evidence indicates that it has a significant intracranial pressure-lowering effect in rodents.^8^ Acute exacerbation of headache often leads to reinvestigation with lumbar puncture, and the collective expert opinion reflected that lumbar puncture provides only temporary relief, can lead in some to longer term complications^9^ and exacerbation of headache.^10^ In those with acute exacerbation of headache, optic nerve examination is essential, and in those found not to have papilloedema, investigation with lumbar puncture and brain imaging is not required, so long as no other secondary causes of headache are suspected. The infographic illustrates the management of acute exacerbation of headache in IIH (figure 1).

Horizon scanning for IIH shows that research is active and that metabolic concepts may potentially provide more understanding of the cause and provide evidence for innovative therapeutic opportunities.^11^ A phase 2 randomised control trial with the first novel drug treatment for IIH has finished recruitment^12^; a phase 3 randomised control trial investigating the best method for weight loss is underway^13^; other surgical trials are in planning.

This infographic highlights three areas that are covered by the consensus guideline for adult IIH, which are: (1) investigation of papilloedema and diagnosis of IIH; (2) management strategies; and (3) investigation and management of acute exacerbation of headache in established IIH^4^ (figure 1).

**Key points**

- Cerebral venography is an essential part of the work-up to exclude venous sinus thrombosis as a cause of papilloedema.
- Lumbar puncture opening pressure forms part of the diagnostic criteria; however, most clinicians feel there is a ‘grey zone’ between 25 cmCSF and 30 cmCSF, which may not be pathological.
- Those with fulminant or precipitous visual decline need urgent surgical treatment, preferably with a ventriculoperintoneal shunt.
- All patients diagnosed with idiopathic intracranial hypertension need sensitive and appropriate discussion regarding weight loss (the only disease-modifying treatment).
- Those with acute exacerbation of headache do not require further neuroimaging or repeat lumbar puncture, unless there are red flag symptoms/signs of infection, or papilloedema with precipitous visual decline.

## References

[1] Mollan SP, Ali F, Hassan-Smith G, et al Evolving evidence in adult idiopathic intracranial hypertension: pathophysiology and management. *J Neurol Neurosurg Psychiatry* 2016;87:982–92. 10.1136/jnnp-2015-311302 26888960 PMC5013119

[2] Markey KA, Mollan SP, Jensen RH, et al Understanding idiopathic intracranial hypertension: mechanisms, management, and future directions. Lancet Neurol 2016;15:78–91. 10.1016/S1474-4422(15)00298-7 26700907

[3] Mollan SP, Markey KA, Benzimra JD, et al A practical approach to, diagnosis, assessment and management of idiopathic intracranial hypertension. Pract Neurol 2014;14:380–90. 10.1136/practneurol-2014-000821 24809339 PMC4251443

[4] Mollan SP, Davies B, Silver NC, et al Idiopathic intracranial hypertension: consensus guidelines on management. J Neurol Neurosurg Psychiatry 2018 10.1136/jnnp-2017-317440 PMC6166610 29903905

[5] Scotton WJ, Mollan SP, Walters T, et al Characterising the patient experience of diagnostic lumbar puncture in idiopathic intracranial hypertension: a cross-sectional online survey. BMJ Open 2018;8:e020445 10.1136/bmjopen-2017-020445 PMC5988086 29848770

[6] Mulla Y, Markey KA, Woolley RL, et al Headache determines quality of life in idiopathic intracranial hypertension. J Headache Pain 2015;16:45 10.1186/s10194-015-0521-9 PMC4436432 25982204

[7] Sinclair AJ, Burdon MA, Nightingale PG, et al Low energy diet and intracranial pressure in women with idiopathic intracranial hypertension: prospective cohort study. BMJ 2010;341:c2701 10.1136/bmj.c2701 20610512 PMC2898925

[8] Scotton WJ, Botfield HF, Westgate CS, et al Topiramate is more effective than acetazolamide at lowering intracranial pressure. Cephalalgia 2018;1:033310241877645 10.1177/0333102418776455 PMC6376637 29898611

[9] Engelborghs S, Niemantsverdriet E, Struyfs H, et al Consensus guidelines for lumbar puncture in patients with neurological diseases. Alzheimers Dement 2017;8:111–26. 10.1016/j.dadm.2017.04.007 PMC5454085 28603768

[10] Yiangou A, Mitchell J, Markey KA, et al Therapeutic lumbar puncture for headache in idiopathic intracranial hypertension: Minimal gain, is it worth the pain? Cephalalgia 2018;1:033310241878219 10.1177/0333102418782192 PMC6376596 29911422

[11] Hornby C, Mollan SP, Botfield H, et al Metabolic concepts in idiopathic intracranial hypertension and their potential for therapeutic intervention. J Neuroophthalmol 2018 doi: 10.1097/WNO.0000000000000684 [Epub ahead of print 6 Jul 2018]. 10.1097/WNO.0000000000000684 PMC6215484 29985799

[12] Markey KA, Ottridge R, Mitchell JL, et al Assessing the Efficacy and Safety of an 11β-Hydroxysteroid Dehydrogenase Type 1 Inhibitor (AZD4017) in the Idiopathic Intracranial Hypertension Drug Trial, IIH:DT: clinical methods and design for a phase II randomized controlled trial. JMIR Res Protoc 2017;6:e181 10.2196/resprot.7806 28923789 PMC5625129

[13] Ottridge R, Mollan SP, Botfield H, et al Randomised controlled trial of bariatric surgery versus a community weight loss programme for the sustained treatment of idiopathic intracranial hypertension: the Idiopathic Intracranial Hypertension Weight Trial (IIH:WT) protocol. BMJ Open 2017;7:e017426 10.1136/bmjopen-2017-017426 PMC5623580 28963303

